# Treatment of Chronic Hepatitis C Virus Infection in Dialysis Patients: An Update

**DOI:** 10.1155/2010/267412

**Published:** 2010-09-20

**Authors:** Hugo Weclawiak, Nassim Kamar, Abdellatif Ould-Mohamed, Isabelle Cardeau-Desangles, Jacques Izopet, Lionel Rostaing

**Affiliations:** ^1^Department of Nephrology, Dialysis, and Organ Transplantation, Toulouse University Hospital, CHU Rangueil, 1 avenue Jean Poulhès, TSA 50032- 31059- Toulouse Cédex 9, France; ^2^INSERM U858, Toulouse University Hospital, CHU Rangueil, IFR 31, 1 avenue Jean Poulhès, TSA 50032, 31059 Toulouse Cédex 9, France; ^3^Laboratory of Virology, Toulouse University Hospital, CHU Purpan, 330 avenue de Grande-Bretagne, TSA 40031, 31059 Toulouse Cédex 9, France; ^4^INSERM U563, Toulouse University Hospital, CHU Purpan, IFR 30, 330 avenue de Grande-Bretagne, TSA 40031, 31059 Toulouse Cédex 9, France

## Abstract

Hepatitis C virus (HCV) infection is a blood-borne infection and its prevalence used to be elevated in hemodialysis (HD) patients. Its main mode of contamination relies on nosocomial transmission. HCV infection is frequently associated in HD patients with normal liver enzymes whereas liver histology can display some degree of HCV-related lesions. The assessment of HCV-related lesions, even in HD dialysis patients, can be done via noninvasive tests. After kidney transplantation, HCV-related lesions can worsen; however, in this setting antiviral treatment harbors the risk of acute rejection. Therefore, it is recommended to implement antiviral treatment while the patient is receiving dialysis therapy. In this setting, the rate of viral clearance is usually high. In case of sustained virological response, no relapse occurs after kidney transplantation, despite heavy immunosuppression.

## 1. Introduction

The most important forms of liver disease in dialysis patients are viral hepatitis B (HBV) and C (HCV). The vast majority of literature on dialysis for hepatitis refers to hemodialysis (HD). Individuals receiving peritoneal dialysis (PD) are at less risk of acquiring blood-borne infections for several reasons, including an absence of extracorporeal blood manipulation, a lack of intravascular access, as well as a lower requirement for blood transfusions. Also, PD takes place in the patient's home, where there is no exposure to other patients.

An accurate assessment of the natural history of HCV in dialysis patients is not easy to obtain. HCV infection in dialysis patients is often asymptomatic with an apparent indolent course. HCV infection extends over decades rather than years whereas chronic kidney-disease (CKD) patients generally have higher morbidity and mortality rates than those of the general population, due to age and comorbidity conditions [1]. This makes the long-term consequences of HCV difficult to establish. Additional factors also modify the course of liver disease, including HBV/HCV coinfection, coinfection with human immunodeficiency virus, or alcohol abuse.

Because of the wide use of antiviral drugs and because posttransfusional hepatitis no longer occurs, future natural-history studies on chronic HCV will become less possible [2]. Accurate evaluation of HCV infection in the CKD population is further complicated by the observation that aminotransferase values are typically lower in dialysis than nonuremic populations [3]. However, dialysis patients that do show detectable HCV RNA have aminotransferase levels greater than those who do not, although values are typically within the “normal” range [4, 5]. Therefore, if one wants to assess the impact of chronic HCV infection in CKD patients, a liver biopsy is usually performed [2]. However, a liver biopsy may be replaced by noninvasive tests, such as a FibroTest or a FibroScan [6, 7], and these tests are of particular interest when there is a possibility of kidney transplantation to treat CKD.

## 2. HCV-Related Outcomes in the CKD Population

A recent meta-analysis on the impact of HCV on mortality in 11,589 maintenance-dialysis patients, from seven observational studies, concluded that the estimated adjusted relative risk (aRR: all cause mortality) was 1.34 (1.13–1.59; *P* < .01) [8]. The cause of death as hepatocellular carcinoma and the incidence of liver cirrhosis, were significantly more frequent among anti-HCV-positive than anti-HCV-negative dialysis patients in all seven studies. The unadjusted summary estimate for liver-related mortality was 5.89 (1.93–17.99; *P* < .001) according to a random-effects model [8].

Recently, Kalantar-Zadeh et al. [9] evaluated a database of 13,664 chronic HD patients in the United States who had undergone HCV serology. They observed that the mortality-hazard ratio was strongly associated with HCV infection: that is, it was 1.25 (1.12–1.39; *P* < .001). Thus, when HCV-positive CKD patients undergo kidney transplantation, it is possible that the natural course of chronic HCV infection is altered by the use of chronic immunosuppression. Indeed, two studies have shown that survival was significantly improved in HCV-positive patients who had benefited from a kidney transplant compared to those who remained on a kidney waiting list [10, 11].

After kidney transplantation, within the first 5 years posttransplant, patient survival is similar in both HCV-positive and HCV-negative patients [12–14]. However, when 10-year survival rates are examined, HCV then appears to be a detrimental effect [12–14]. A meta-analysis of observational studies identified eight clinical trials (6,365 unique patients) in which the presence of anti-HCV antibodies in the serum was an independent and significant risk factor for death and graft failure after kidney transplantation. The estimates for relative risk (RR) were 1.79 (1.57–2.03) and 1.56 (1.35–1.80), respectively [15].

The adverse impact of HCV on survival after kidney transplantation has been linked to liver dysfunction. Furthermore, a positive anti-HCV serology in kidney-transplant patients has been implicated in the development of *de novo* glomerulopathy [19], an increased incidence of serious infections [20], and new-onset diabetes mellitus [21]. In addition, in kidney-transplant patients, the use of alpha-interferon (*α*IFN) to treat HCV infection has been associated with (i) a poor response to antiviral therapy and (ii) the occurrence of a high rate of acute rejection, that is, up to 50% in some series [22, 23]. The latter were mainly humoral (sub)acute rejections [24]. Conversely, in kidney-transplant patients, the use of pegylated alpha-interferon (peg*α*IFN), although more limited, has been rarely associated with acute allograft rejection [25]. Because of the above concerns, it seems reasonable to treat HCV infection while the patient is on dialysis, that is, before they are placed on a kidney-transplant waiting list.

## 3. HCV Treatment in the General Population

At the moment, the best treatment for chronic HCV infection in patients with normal renal function relies on the combined use of peg*α*IFN and ribavirin (RBV). Thus, Hartwell and Shepherd recently performed a meta-analysis that included ten randomized, controlled trials (RCTs) in which treatment was based on peg*α*IFN/RBV or *α*IFN/RBV [26]. peg*α*IFN/RBV therapy resulted in significantly higher sustained virological response (SVR) rates than treatment with the combined *α*IFN/RBV therapy. Treatment for 48 weeks with peg*α*IFN/RBV was significantly more effective than the same treatment for 24 weeks. Significantly higher SVR rates were seen with combined *α*IFN/RBV compared to either an *α*IFN monotherapy or to no treatment. In this meta-analysis (four *α*IFN trials), the relative risk of not experiencing an SVR was 0.59 (95% CI, 0.51–0.69) and was highly statistically significant (*P* < .00001). SVRs were higher for patients with genotype non-1 compared with genotype 1 for both peg*α*IFN/RBV and IFN/RBV treatments [26].

## 4. HCV Treatment in the CKD Population

The AASLD (American Association for the Study of Liver Diseases) has published guidelines for the CKD population stating that “when HCV infection is identified in persons with CKD, interferon-based antiviral treatment must be considered, but the regimen will vary depending of the kidney disease…The decision to treat must take into account the competing severities of the CKD and the chronic liver disease, the risks of the treatment itself,…, and whether there are comorbid conditions that may affect morbidity and mortality, such as cardiovascular disease.” [27].

The kidneys play a major role in the catabolism and filtration of both interferon and ribavirin; thus, their clearances may be affected in subjects with CKD [28, 29]. The clearance of pegylated interferon is affected in those with CKD, although hemodialysis does not affect its clearance [30]. Hence, the AASLD guidelines recommend subcutaneous weekly doses of 1 *μ*g/kg of peginterferon alpha-2b and of 135 *μ*g of peginterferon alpha-2a [27] to patients with stage 3–5 CKD. Because ribavirin is eliminated by the kidney, and if overdosed might result in dramatic anemia [31], ribavirin therapy is contraindicated when creatinine clearance is <50 mL/min. Hence, most data regarding HCV treatment in the CKD population deal with the use of either standard *α*-interferon or *α*-pegylated interferon.

## 5. Treatment of Chronic HCV Infection in CKD Patients

With regards to end-stage kidney-disease (ESKD) patients who are chronically treated by dialysis, Casanovas-Taltavull et al. reviewed two meta-analyses (Meta-1 and Meta-2) published in 2008 (Table 1). From these, they analyzed the SVRs, any adverse effects, and the reasons for discontinuing *α*IFN treatment in dialysis patients [32]. The Meta-1 study analyzed results obtained from 645 patients; the Meta-2 study used data from 459 patients (19 studies were duplicated). Overall, the SVR was 40%; SVR in genotype 1 was 33%, with pegylated interferon providing few additional benefits over conventional alpha-interferon. Adverse events, such as typical flu-like syndrome occurred in 41% of patients, requiring withdrawal of antiviral treatment in 11% of them. A high rate of anemia was also documented, although the use of recombinant erythropoietin, intravenous iron administration, or transfusions was not generally reported. A typical flu-like syndrome occurred in 41% of patients, which required withdrawal of antiviral treatment in 11%. Severe adverse events were divided into the following groups: hormonal (thyroid), bone pain, cytopenia, gastrointestinal, immunological (prior graft rejection), central nervous system, cardiovascular, and infectious problems. The reviewers of these meta-analyses pointed out any bias in the selection criteria of candidates for treatment, limitations related to the number and type of adverse effects (as well as their clinical evaluation), and discrepancies in cases of discontinuation of treatment or loss to follow-up.

With regards to Meta-1, the primary outcome was a SVR (as a measure of efficacy); the secondary outcome was the drop-out rate (as a measure of tolerability) [16]. They identified 13 prospective studies, which were controlled clinical trials that included 539 unique patients, of whom 10 (76.9%) patients were receiving maintenance dialysis. Pooling of these studies' results showed a significant increase in viral response of patients treated with antiviral therapy compared to patients who did not receive any therapy (controls). The pooled odds ratio (OR) of failure to obtain a SVR was 0.081 (95% confidence intervals (CI), 0.029–0.230), *P* = .0001. The pooled OR of drop-out rate was significantly increased in treated versus control patients, OR = 0.389 (95% CI, 0.155–0.957), *P* = .04. The studies were heterogeneous with regard to viral response and drop-out rate. In the subset of clinical trials (*n* = 6) involving only dialysis patients receiving *α*-IFN monotherapy for chronic HCV, there was a significant difference in the risk of failure to obtain a SVR (study versus control patients), OR = 0.054 (95% CI, 0.019; 0.150), *P* = .0001. No difference in the drop-out rate between study and control patients was shown (OR = 0.920 (95% CI, 0.367; 2.311), NS). Meta-1 showed that viral response was greater in patients with chronic kidney disease who received antiviral therapy than in controls. No differences in the drop-out rates between study and control patients occurred in the subgroup of dialysis patients on *α*-IFN monotherapy [16].

With regards to Meta-2, the authors took into account those chronic dialysis patients with chronic HCV infection who were either treated with *α*IFN or peg*α*IFN, with or without ribavirin [17]. They searched on MEDLINE for indexed studies since 1966, and only selected studies with a sample size greater than 10. They looked for the following parameters: SVR at 6 months after treatment, rate of treatment discontinuation caused by adverse events, and factors associated with these outcomes. They analyzed 20 studies that contained 459 *α*IFN-treated patients, three studies that contained 38 peg*α*IFN-treated patients, and two studies that contained 49 peg*α*IFN and ribavirin-treated patients. The overall SVR rate was 41% (95% confidence interval [CI], 33 to 49) for *α*IFN and 37% (95% CI, 9 to 77) for peg*α*IFN. Treatment-discontinuation rates were 26% (95% CI, 20 to 34) for *α*IFN and 28% (95% CI, 12 to 53) for peg*α*IFN. SVR was higher, with 3 million units (MU) or higher of *α*IFN at three times weekly, with lower mean amounts of HCV RNA, lower rates of cirrhosis, a HCV genotype 1, or elevated transaminase, though these findings were not statistically significant.

Treatment-discontinuation rates were greater in studies using larger doses. Hence, side-effects from alpha-interferon were numerous, particularly in the ESKD population. The main side effects were fatigue/weakness and loss of appetite, which may lead to weight loss and, thus, fluid overload if dry weight is not adapted accordingly. Many patients also developed anemia, which often requires commencement or increased treatment with erythropoietin-stimulating agents; in addition, seizures can occur if there is fluid overload and hypertension. The limitations of these meta-analyses were publication bias, there were few randomized controlled trials, and there were limitations in generalizability of all hemodialysis patients. In conclusion, alpha-IFN treatment of hemodialysis patients resulted in an SVR rate of 41%. Thus, a higher weekly dose of *α*IFN, a lower mean level of pretreatment HCV RNA, a lower rate of cirrhosis, an HCV genotype different from 1, and/or decreased transaminase levels may all be associated with greater SVR rates [17].

A more recent meta-analysis has been published on a group of 770 hemodialysis patients with chronic HCV infection (Table 1), in which the authors evaluated factors that were associated to SVR after *α*-pegylated or standard *α*-IFN monotherapy. Twenty-one studies on *α*-IFN-alfa2a or *α*-IFN-alfa2b (491 patients) and 12 on pegylated-IFN-alfa2a or PEG-IFN-alfa2b (279 patients) were evaluated. The pooled SVRs for standard and pegylated *α*-IFN monotherapy in random-effect models were 39.1% (95% CI, 32.1 to 46.1) and 39.3% (95% CI, 26.5 to 52.1), respectively. Pooled dropout rates were 22.6% (95% CI, 10.4 to 34.8) and 29.7% (95% CI, 21.7 to 37.7), respectively. Female gender, HCV-RNA copies per mL, HCV genotype, alanine transaminase pattern, duration of infection, stage of liver fibrosis, and treatment duration were not associated with SVR. Only an age less than 40 years was significantly associated with SVR (odds ratio, 2.17; 95% CI, 1.03 to 4.50) [18].

There are only few limited reports that describe the combined use of (peg)alpha-interferon and ribavirin in dialysis patients. With regard to this combined therapy, the AASLD guidelines state that “Ribavirin can be used in combination with interferon with a markedly reduced daily dose with careful monitoring for anemia and other adverse effects.” [27]. The largest series published so far on the combined use of peginterferon alpha-2a plus ribavirin in hemodialysis patients obtained a SVR rate of 97% (34/35) in the treated patients (peginterferon alpha-2a plus ribavirin) versus 0% (0/35) in untreated controls [33]. These findings have not been confirmed in further reports where the SVR rate ranges between 7% and 71% [1].

## 6. Treatment of Acute HCV Infection in CKD Patients

In the general population, with regard to the treatment of acute HCV infection, the AASLD guidelines state that “Treatment can be delayed for 8 to 12 weeks after acute onset of hepatitis to allow spontaneous resolution; …Although excellent results were achieved using standard interferon monotherapy, it is appropriate to consider the use of peginterferon…Until more information becomes available, no definitive recommendation can be made about the optimal duration needed for treatment of acute hepatitis C; however, it is reasonable to treat for at least 12 weeks and 24 weeks may be considered.” [27].

In dialysis patients, Liu et al. have very recently published their experience regarding the treatment of acute HCV infection. They included 35 dialysis patients that had no spontaneous clearance of HCV at 16 weeks after acute HCV infection. They were thus then given a course of peginterferon alpha 2a at 135 *μ*g weekly for 24 weeks [34]. They compared the results with those from a historical series of 36 hemodialysis patients who had acute hepatitis C, but had not received treatment. The rate of SVR in their treatment group was significantly higher than the rate of spontaneous HCV clearance in the control historical series group (88.6% versus 16.7%). All but one patient had a rapid virologic response (undetectable HCV RNA levels at 4 weeks of therapy), and all patients who received more than 12 weeks of therapy had early and end-of-treatment virologic responses. All patients who had clearance of HCV by 16 weeks had undetectable HCV RNA levels during and at the end of follow-up. Liu et al. conclude that “Pegylated IFN alfa-2a monotherapy is safe and efficacious for hemodialysis patients with acute hepatitis C. It is suggested that patients without spontaneous clearance of HCV by week 16 should receive this therapy.” [34].

In addition, dialysis patients who were cleared of the HCV virus after antiviral therapy, and received kidney transplantation, did not present with HCV reactivation, despite heavy immunosuppression [35]. Hence, 16 HCV seropositive/HCV RNA-positive hemodialysis patients who were treated with IFN-alpha (9 MU/wk during 6 or 12 months) underwent kidney transplantation 38 months (range: 2 to 57) after alpha-IFN therapy. At kidney transplantation, HCV viremia was negative in all patients. Immunosuppression relied on anticalcineurin agents with or without steroids and/or antimetabolites; in addition, 12 of them received induction therapy with antithymocyte globulins; at the last follow-up after kidney transplantation, that is, 22.5 months (range, 2 to 88), HCV viremia remained negative in all patients [35]. Recently, we have assessed the persistence of HCV infection in 26 HCV seropositive kidney-transplant patients currently receiving immunosuppressants, and who were formerly infected with HCV, that is, they had eliminated HCV either spontaneously or after interferon-*α* therapy while on hemodialysis [36]. No biochemical or virological relapse was seen during the median posttransplant follow-up of 10.5 years (range: 2–16) in those patients who received immunosuppressive therapy that included calcineurin inhibitors (100%), and/or steroids (62%), and/or antimetabolites (94%). At the last follow-up, all had undetectable HCV RNA according to the conventional tests that were repeated, on average, five times (range, 1–15). We also looked for residual HCV RNA in their plasma and peripheral blood-mononuclear cells (PBMCs) (stimulated or not in culture) with an ultrasensitive RT-PCR assay, followed by Southern blotting for PBMCs: no HCV genomic RNA was detected in the plasma samples or in the unstimulated and stimulated PBMCs. Thus, an absence of a relapse of HCV in formerly HCV-infected immunocompromised patients suggests complete eradication of HCV after its elimination while on dialysis [36]. These findings highlight the fact that HCV-positive dialysis patients who have a SVR after completion of alpha-(peg)interferon therapy are really cured of HCV.

We conclude that, because it is not always safe to treat HCV infection after kidney transplantation, antiviral treatment should be implemented before transplantation, that is, while the patient is on dialysis therapy. The evidence suggests that treatment might be based on alpha interferon (standard or pegylated), this results in a high rate of sustained viral clearance. In cases where there is no virological response, one could add very low doses of ribavirin therapy to the alpha-interferon in order to maximise the virological response. However, one needs to be mindful of the risk of hemolytic anemia. Finally, dialysis HCV seropositive patients who have a sustained virological response after antiviral therapy do not relapse after kidney transplantation despite powerful immunosuppressive therapy.

## Figures and Tables

**Table 1:** Treatment with alpha-interferon or pegylated-alpha interferon in dialysis HCV positive patients: results from 3 meta-analyses.

	Meta-analysis 1 (Fabrizi et al. [16])	Meta-analysis 2 (Gordon et al. [17])	Meta-analysis 3 (Alavian and Tabatabaei; [18])
Number of studies	28	25	33
Number of patients	645	459	770
Overall SVR (standard IFN/Peg-INF) %	39/41	41/37	39.1/39.3
Genotype 1(%)	33	Not reported	Not reported
Treatment discontinuation (standard IFN/Peg-IFN/Placebo) %	19/27/ not reported	26/28/22	22.6/29.7/not reported

SVR: sustained virological response; IFN: alpha-interferon; Peg-IFN: pegylated alpha-interferon.

## References

[1] Fabrizi F, Messa P, Basile C, Martin P (2010). Hepatic disorders in chronic kidney disease. *Nature Reviews Nephrology*.

[2] Kidney Disease: Improving Global Outcomes (2008). KDIGO clinical practice guidelines for the prevention, diagnosis, evaluation, and treatment of hepatitis C in chronic kidney disease. *Kidney International. Supplement*.

[3] Guh J-Y, Lai Y-H, Yang C-Y (1995). Impact of decreased serum transaminase levels on the evaluation of viral hepatitis in hemodialysis patients. *Nephron*.

[4] Salama G, Rostaing L, Sandres K, Izopet J (2000). Hepatitis C virus infection in French hemodialysis units: a multicenter study. *Journal of Medical Virology*.

[5] Fabrizi F, Lunghi G, Andrulli S (1997). Influence of hepatitis C virus (HCV) viraemia upon serum aminotransferase activity in chronic dialysis patients. *Nephrology Dialysis Transplantation*.

[6] Varaut A, Fontaine H, Serpaggi J (2005). Diagnostic accuracy of the fibrotest in hemodialysis and renal transplant patients with chronic hepatitis C virus. *Transplantation*.

[7] Sebastiani G, Halfon P, Castera L (2009). SAFE biopsy: a validated method for large-scale staging of liver fibrosis in chronic hepatitis C. *Hepatology*.

[8] Fabrizi F, Takkouche B, Lunghi G, Dixit V, Messa P, Martin P (2007). The impact of hepatitis C virus infection on survival in dialysis patients: meta-analysis of observational studies. *Journal of Viral Hepatitis*.

[9] Kalantar-Zadeh K, Kilpatrick RD, McAllister CJ (2007). Hepatitis C virus and death risk in hemodialysis patients. *Journal of the American Society of Nephrology*.

[10] Knoll GA, Tankersley MR, Lee JY, Julian BA, Curtis JJ (1997). The impact of renal transplantation on survival in hepatitis C-positive end-stage renal disease patients. *American Journal of Kidney Diseases*.

[11] Pereira BJG, Levey AS (1997). Hepatitis C virus infection in dialysis and renal transplantation. *Kidney International*.

[12] Hanafusa T, Ichikawa Y, Kishikawa H (1998). Retrospective study on the impact of hepatitis C virus infection on kidney transplant patients over 20 years. *Transplantation*.

[13] Legendre CH, Garrigue V, Le Bihan C (1998). Harmful long-term impact of hepatitis C infection in kidney transplant recipients. *Transplantation*.

[14] Mathurin P, Mouquet C, Poynard T (1999). Impact of hepatitis B and C virus on kidney transplantation outcome. *Hepatology*.

[15] Fabrizi F, Martin P, Dixit V, Bunnapradist S, Dulai G (2005). Hepatitis C virus antibody status and survival after renal transplantation: meta-analysis of observational studies. *American Journal of Transplantation*.

[16] Fabrizi F, Ganeshan SV, Lunghi G, Messa P, Martin P (2008). Antiviral therapy of hepatitis C in chronic kidney diseases: meta-analysis of controlled clinical trials. *Journal of Viral Hepatitis*.

[17] Gordon CE, Uhlig K, Lau J, Schmid CH, Levey AS, Wong JB (2008). Interferon treatment in hemodialysis patients with chronic hepatitis C virus infection: a systematic review of the literature and meta-analysis of treatment efficacy and harms. *American Journal of Kidney Diseases*.

[18] Alavian SM, Tabatabaei SV (2010). Meta-analysis of factors associated with sustained viral response in patients on hemodialysis treated with standard or pegylated interferon for hepatitis C infection. *Iranian Journal of Kidney Diseases*.

[19] Morales JM, Campistol JM, Dominguez-Gil B (2002). Hepatitis C virus infection and kidney transplantation. *Seminars in Nephrology*.

[20] Bouthot BA, Murthy BVR, Schmid CH, Levey AS, Pereira BJG (1997). Long-term follow-up of hepatitis C virus infection among organ transplant recipients: implications for policies on organ procurement. *Transplantation*.

[21] Kamar N, Mariat C, Delahousse M (2007). Diabetes mellitus after kidney transplantation: a French multicentre observational study. *Nephrology Dialysis Transplantation*.

[22] Rostaing L, Modesto A, Baron E, Cisterne JM, Chabannier MH, Durand D (1996). Acute renal failure in kidney transplant patients treated with interferon alpha 2b for chronic hepatitis C. *Nephron*.

[23] Thervet E, Pol S, Legendre C, Gagnadoux M-F, Cavalcanti R, Kreis H (1994). Low-dose recombinant leukocyte interferon-*α* treatment of hepatitis C viral infection in renal transplant recipients: a pilot study. *Transplantation*.

[24] Baid S, Tolkoff-Rubin N, Saidman S (2003). Acute humoral rejection in hepatitis C-infected renal transplant recipients receiving antiviral therapy. *American Journal of Transplantation*.

[25] Pageaux G-P, Hilleret M-N, Garrigues V (2009). Pegylated interferon-*α*-based treatment for chronic hepatitis C in renal transplant recipients: an open pilot study. *Transplant International*.

[26] Hartwell D, Shepherd J (2009). Pegylated and non-pegylated interferon-alfa and ribavirin for the treatment of mild chronic hepatitis C: a systematic review and meta-analysis. *International Journal of Technology Assessment in Health Care*.

[27] Ghany MG, Strader DB, Thomas DL, Seeff LB (2009). Diagnosis, management, and treatment of hepatitis C: an update. *Hepatology*.

[28] Bocci V, Pacini A, Muscettola M (1981). Renal filtration, absorption and catabolism of human alpha interferon. *Journal of Interferon Research*.

[29] Glue P (1999). The clinical pharmacology of ribavirin. *Seminars in Liver Disease*.

[30] Gupta SK, Pittenger AL, Swan SK (2002). Single-dose pharmacokinetics and safety of pegylated interferon-*α*2b in patients with chronic renal dysfunction. *Journal of Clinical Pharmacology*.

[31] Brochot E, Castelain J, Duverlie G (2010). Ribavirin monitoring in chronic hepatitis C therapy: anaemia versus efficacy. *Antiviral Therapy*.

[32] Casanovas Taltavull T, Baliellas Comellas C, Cruzado Garrit JM (2009). Results of hepatitis C virus treatment in patients on hemodialysis: data from published meta-analyses in 2008. *Transplantation Proceedings*.

[33] Rendina M, Schena A, Castellaneta NM (2007). The treatment of chronic hepatitis C with peginterferon alfa-2a (40 kDa) plus ribavirin in haemodialysed patients awaiting renal transplant. *Journal of Hepatology*.

[34] Liu CH, Liang CC, Liu CJ (2010). Pegylated interferon alfa-2a monotherapy for hemodialysis patients with acute hepatitis C. *Clinical Infectious Diseases*.

[35] Kamar N, Toupance O, Buchler M (2003). Evidence that clearance of hepatitis C virus RNA after *α*-interferon therapy in dialysis patients is sustained after renal transplantation. *Journal of the American Society of Nephrology*.

[36] Nicot F, Kamar N, Mariamé B, Rostaing L, Pasquier C, Izopet J (2010). No evidence of occult hepatitis C virus (HCV) infection in serum of HCV antibody-positive HCV RNA-negative kidney-transplant patients. *Transplant International*.

